# The Object Orientation Effect in Exocentric Distances

**DOI:** 10.3389/fpsyg.2018.01374

**Published:** 2018-08-03

**Authors:** Marlene Weller, Kohske Takahashi, Katsumi Watanabe, Heinrich H. Bülthoff, Tobias Meilinger

**Affiliations:** ^1^Max Planck Institute for Biological Cybernetics, Tübingen, Germany; ^2^School of Psychology, Chukyo University, Nagoya, Japan; ^3^Department of Intermedia Art and Science, Waseda University, Tokyo, Japan

**Keywords:** distance perception, object orientation, exocentric distance, action preparation, predictive coding

## Abstract

The object orientation effect describes shorter perceived distances to the front than to the back of oriented objects. The present work extends previous studies in showing that the object orientation effect occurs not only for egocentric distances between an observer and an object, but also for exocentric distances, that are between two oriented objects. Participants watched animated virtual humans (avatars) which were either facing each other or looking away, and afterward adjusted a bar to estimate the perceived length. In two experiments, participants judged avatars facing each other as closer than avatars facing away from each other. As the judged distance was between two objects and did not involve the observer, results rule out an explanation that observers perceive object fronts as closer to prepare for future interaction with them. The second experiment tested an explanation by predictive coding, this is the extrapolation of the current state of affairs to likely future states here that avatars move forward. We used avatars standing on bridges either connecting them or running orthogonal to the inter-avatar line thus preventing forward movement. This variation of walkability did not influence participants’ judgments. We conclude that if predictive coding was used by participants, they did not consider the whole scene layout for prediction, but concentrated on avatars. Another potential explanation of the effect assumes a general asymmetrical distribution of inter-person distances: people facing each other might typically be closer to each other than when facing away and that this asymmetry is reflected as a bias in perception.

## Introduction

The importance of objects around us is depending, inter alia, on their distance to us. In keeping and perceiving distance, we do not only rely on pure sensory information but we also consider various factors such as the expected size of an object (McIntosh and Lashley, 2008). The factor we are analyzing in this study is object orientation.

People keep a greater distance to a virtual human character’s front than to its back (Bailenson et al., 2003). They also judge avatars as closer if they face those (Jung et al., 2016). This object orientation effect on distance perception is also found for virtual cones and cameras (Takahashi et al., 2013; Foster et al., unpublished). So, in the egocentric perspective, humans are aware of being faced and adapt their behavior accordingly.

Where does the object orientation effect on distance perception come from? Potential explanations might be based on: (1) social and emotional factors, (2) preparation for action in general or (3) extrapolations of forward oriented movements and locations. In accordance with previous research on social and emotional influences on perception, such influences might constitute the object orientation effect. For example, if we feel “close” to somebody, we feel comfortable at shorter distances (Willis, 1966; Patterson, 1977), the opposite applies to people we dislike (Kleck, 1968) or which are characterized as immoral (Iachini et al., 2015). We also perceive the distance to desired (Balcetis and Dunning, 2010) or threatening objects (Cole et al., 2013) as closer as to undesired or neutral objects. These arguments are consistent with a social origin of the object orientation effect. However, there are some reasons speaking against social or emotional factors. Firstly, the effect is independent of gaze. Gaze is an important social cue (Tomasello et al., 2007). But only body orientation influenced distant perception significantly and being gazed at vs. not did not influence distance perception (Jung et al., 2016). Furthermore, the effect is also found with objects (Takahashi et al., 2013; Foster et al., unpublished). Takahashi et al. used animate cones pointing toward participants or away from them. Perceiving the cones’ tips as dangerous and frightening or interpreting the cones as living beings, might have constituted the effect. However, Foster et al. (unpublished) ruled out these possibilities: they used non-aversive objects, namely cameras, which ruled out fear as a mediator. They replicated the effect with static cameras ruling out perception of animation as a prerequisite. Consequently, social and emotional factors do not seem to constitute the effect.

Various accounts describe how perception is shaped in the light of potential actions (Gibson, 1979; Proffitt, 2006; Witt, 2017). So, perceived distance is adapted to our capabilities. For example, if people carry a heavy backpack, a hill looks steeper, or if people are afraid of fall heights are judged as higher (Proffitt et al., 2003; Proffitt, 2006; for a critical reception see Firestone and Scholl, 2016). The perceived distance increases with the cost or effort of an action, and therefore, indicates whether an action is within our capabilities.

The near space within which we can interact without moving the whole body is represented differently than more distant space. Rizzolatti et al. (1997) examined how maps of objects lying within the peripersonal space are represented in the ventral premotor cortex. Objects that are close at hand are mostly represented in bimodal areas “responding both to visual three-dimensional stimuli and to tactile stimuli” (Rizzolatti et al., 1997). If people can use a tool to reach more distant objects, the peripersonal space will increase (Berti and Frassinetti, 2000). This mapping could reflect potential interaction.

In this sense, objects facing an observer may look closer if they afford interaction than if they are oriented away. If the object orientation effect depends on a perceptual bias due to potential interactions with an object, it has to be limited to the egocentric distances between the observer and the object. Allocentric distances between objects facing each other will not afford an observer’s interaction with them. Consequently, object orientation should not affect the perceived distance between two objects. In case the object orientation effect is found anyway, this would indicate that it is not based on perceptual distortions due to potential interaction with the avatar. Experiment 1 tested this prediction. Observers judged the distance between two avatars which were either facing each other or which were facing away. The results of Experiment 1 showed that the object orientation effect also exists in exocentric perspective. Therefore, the object orientation effect is not due to preparation for future interactions.

Experiment 2 looked into another potential explanation of the object orientation effect. That is predictive coding (Kilner et al., 2007; Clark, 2013). Brains are considered to be “prediction machines” (Clark, 2013) whose aim is to minimize the error between predictions about the world and actual sensory input. Predicting changes and further states successfully provide advantages to respond to the environment reasonably. When we watch other people, we automatically predict their further actions. Objects with a front typically move toward their front rather than toward other orientations. This is true for most animals as well as artifacts, such as screws, cars, bikes, etc. Extrapolating their location toward their front would contribute to their likely future state and might thus support better predictions about the world. An explanation based on predictive coding would not be limited to exocentric distances but also expect an object orientation effect for allocentric distances between objects. However, depending on the complexity of the prediction, such an effect might be influenced by how likely such a movement is. Ensuing from the result of Experiment 1, which showed the effect in an allocentric perspective, we restricted the possibilities of actions of the two avatars in Experiment 2. We used virtual bridges to manipulate the possible movements of the avatars. If the avatars stood on the same bridge, it was possible for them to walk toward each other or away from each other. In the second condition, they were standing on two parallel bridges with a gap between them. The gap was preventing them from getting closer to each other. If the perception depends on predictive coding, we expect that the number of bridges interacts with the orientation of the avatars. When they stood on the same bridge, we expected that the estimated distance between avatars facing each other to be perceived as smaller as the distance between avatars standing back to back. When they stood on different bridges with a gap too wide to jump over, we expected no difference in the error for the two orientations. In the case the object orientation effect is still observable in the condition with two bridges we do not have to reject the theory of predictive coding as the scene is quiet complex and maybe not be sufficient enough to prevent forward extrapolation.

## Experiment 1

### Materials and Methods

#### Participants

Twenty volunteers (10 females) participated after giving informed written consent and were paid 8 Euros per hour. One female was excluded due to errors in data collection. The mean age was 25.74 years (*SD* = 5.26 years). The study was approved by the ethics committee of the University Clinic Tübingen.

#### Setup

During the experiment participants sat on a rotatable chair which approximately maintained their natural body height. They used a game controller to operate the experiment. To display the virtual reality, we used the head-mounted display Oculus Rift Development Kit 2. It has a resolution of 960 × 1,080 pixel per eye and a field of view of 100°× 100°. Participants’ head position was tracked by 16 high-speed infrared motion capture cameras with 150 HZ (Vicon^®^ MX 13). A computer rendered an egocentric view of the virtual environment in the head-mounted display in real-time on a NVIDIA GTX1080 graphics card. The experiment was programed in Unity 4.6.5.

#### Stimuli and Trial

**FIGURE 1:**
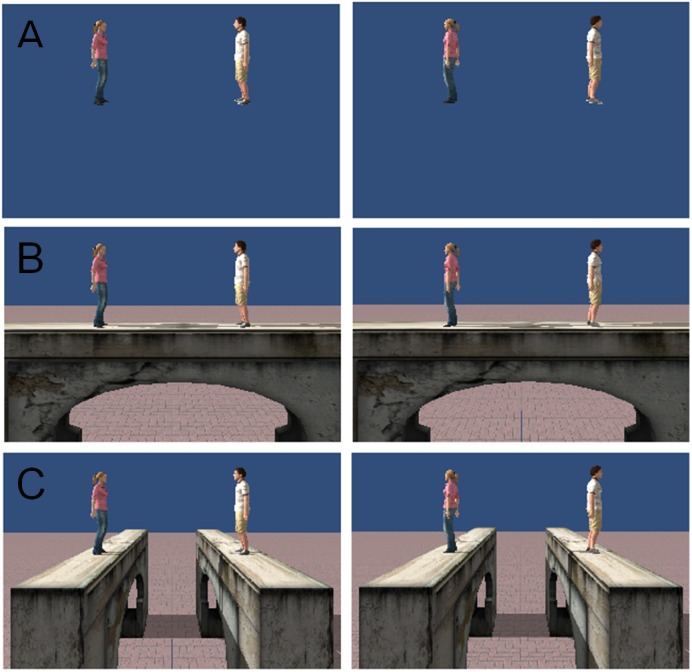
Static snapshots from the experimental conditions. In Experiment 1, avatars were presented in free space **(A)**. In Experiment 2, avatars were presented on the same bridge **(B)** or on parallel bridges **(C)**.

We used two virtual human characters (“avatars”), one male and the other female, from Rocketbox^TM^, just as in previous experiments (Jung et al., 2016). The male avatar had a height of 175 cm and the female 165 cm. During their presentation, the avatars were animated with the same animation. They swayed slightly from left to right to enhance realism. The perceived midpoint of the animated avatars was determined in a pre-experiment (see Jung et al., 2016). The avatars stood on an invisible plane aligned with the physical floor. We varied the gap between the avatars (1–3.5 m in steps of 0.5 m), the distance between avatars and participant (2–8 m in steps of 2 m) and the avatars’ facing direction (either facing each other or facing away from each other) (**Figure 1**).

After watching the avatars for 3 s, participants had to adjust the length of a virtual bar with the joystick of the game controller. The bar appeared 45° to the right. The bar was oriented orthogonal to a participant’s viewing axis at a distance of 6 m and a height of 0.8 m above the (invisible) ground plane. The original length of the bar alternated between 0.2 and 12 m. Participants confirmed their estimated length with a button press, and the bar disappeared. To continue with the next trial, participants had to turn back to the front, look straight ahead to a fixation cross and press a button. They had the opportunity to take a break after every trial. Participants were instructed to act as accurately and quickly as possible. We recorded the estimated length and latency (i.e., time between bar appearance and button press). Latency was not relevant for our purposes.

#### Procedure

First, participants received detailed written and oral description of the task. They trained the task on example trials as long as they wanted, but at least eight times before proceeding to the real experiment. After the experiment, participants filled out a questionnaire. The experiment lasted about 60 min.

#### Design and Analysis

We used a 2 (facing direction of avatars) × 6 (gap between avatars) × 4 (distance to the participant) × 2 (placement of male and female avatar) fully balanced within design. We repeated 96 trials three times, resulting in 288 trials altogether. Each block was presented in random order.

As we were not interested in the avatars’ sex as such, but wanted to guarantee a minimum of diversity of avatar constellations, the avatars’ sex (i.e., their positioning) was not analyzed as an experimental factor. The length of the bar at the beginning of each trial was also not analyzed. However, including avatar sex or bar length into the analyses did not change the pattern of the reported results.

We removed values that deviated more than 3 standard deviations from the overall mean and trials in which the participant did not adjust the bar. We computed an ANOVA for the factor “estimated gap between avatars” with within-subjects factors of distance to the participant (4 levels), gap between avatars (6 levels), and facing direction of avatars (2 levels). If the ANOVA’s assumption of sphericity was violated in any of our experiments, we corrected the degrees of freedom using the Greenhouse-Geisser estimates. The analysis was performed with RStudio.

### Results

Participants judged the distance between two avatars which were either facing each other or looking away from each other. We expected that distances between facing avatars are perceived as shorter.

**FIGURE 2:**
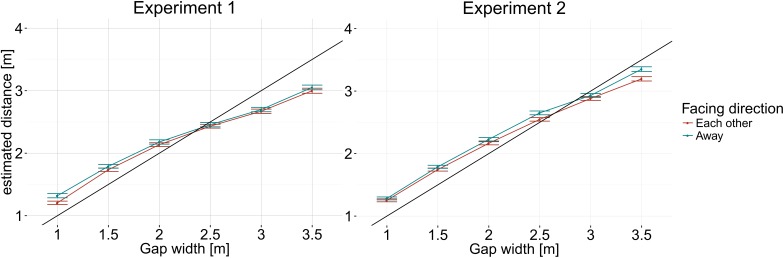
Estimated gap between avatars as a function of actual gap between avatars and avatar facing direction. Means and standard errors as estimated from the marginal means are shown. The diagonal line indicates the correct gap width.

**Figure 2** shows the estimated distances depending on avatar facing direction and width of gap between avatars. Distance estimations differed depending on facing direction of avatars, *F*(1,18) = 16.69, *p* < 0.001. Subjects perceived the gap between avatars as smaller when the two avatars faced each other than when they faced away. We also observed a main effect of gap width, *F*(1.26,22.70) = 35.63, *p* < 0.001. Gap width estimates increased with actual avatar distance. When the gap was small, the gap was overestimated. When the gap was wide, it was underestimated. The smallest error was achieved at a gap width of 2.5 m between avatars.

Furthermore, distance from the participant interacted with gap width, *F*(5.03,90.58) = 5.40, *p* < 0.001. Participants estimated all distances except of the distance of 2 m according to the schema displayed in **Figure 1**. At a distance of 2 m, participants estimated the distance more accurately. No other main effects or interactions attained significance.

### Discussion

People judge avatars (Jung et al., 2016) and objects (Takahashi et al., 2013; Foster et al., unpublished) as closer if they face them rather than if they face away. This study examined whether the explanation for the object orientation effect which is preparation for interaction can be rejected.

When the effect is based on preparation for action (Gibson, 1979; Proffitt, 2006; Witt, 2017), we do not expect an effect for distance estimation for non-egocentric distances. In this experiment, participants judged the distance between two avatars either facing each other or facing away. We observed shorter distance estimations for avatars facing each other than for those facing away. The effect cannot be explained with participants preparing for an interaction with the avatar.

Could a strategy of concentrating on the minimal distance explain the effect? We equalized the distance between the avatars by their perceived midpoint during animation as determined in pre-experiments. When the avatars faced each other, the minimal distance between them (i.e., between their noses) was about 10 cm shorter than the minimal distance between them when they did not faced each other (i.e., between the back of their heads or between their feet). The minimum horizontal distances between any point (i.e., between noses in case of facing and between the back of the women’s head and the men’s behind in case of facing away) were equal. Nevertheless, in case participants used the shortest horizontal distance between the avatar bodies, which is different from the distance between their midpoints, this could explain the main effect observed. Please note, that such a strategy could not explain the effects observed in egocentric distance estimation in the experiments from Jung et al. (2016) and Takahashi et al. (2013). In the later object orientation interacted with distance from the observer (Takahashi et al., 2013). In the experiment of Jung et al. (2016) the avatars rotated around their own axis. According to the minimal distance strategy avatars standing sideways to the observer should have been judged closer compared to avatars facing or facing away. Nevertheless facing avatars were judged as closest.

We observed that for small gap width the distance was overestimated and for wide gap width the distance was underestimated. This effect of regression to the mean can be frequently observed when a fixed range of magnitude estimation test samples is presented (Petzchner et al., 2015). The participants based their judgments not only on the observed stimulus but also on the range of the tested distribution. They adjust the estimated distance to its average.

In the interaction of the factors gap between avatars and distance to participants we can see that the effect of regression was less strong at a distance of 2 m, thus, the estimation was better. Maybe the distance was less underestimated for wide gaps with a short distance to participants, because the participants had to turn their head more in order to fixate an avatar. Additionally, the second avatar was out of sight when the head was turned to the left or the right. Compared to other conditions, such a movement could evoke the perception of a greater distance in participants.

People judge distances between persons as shorter when they face each other than when they face away. This effect is not limited to an egocentric perspective, thus, it is not due to preparation of action. To examine if the effect is due to extrapolations of location in forward direction, we conducted Experiment 2.

## Experiment 2

### Materials and Methods

For Experiment 2, we modified the setup of Experiment 1. In the following only modifications are reported.

#### Participants

Eight women and twelve men took part in the experiment. The mean age was 29.37 years (*SD* = 8.97 years).

#### Stimuli

In Experiment 2 the same avatars and conditions as in Experiment 1 were used, but the avatars were standing on bridges, and a floor was visible. We added the two-factorial condition “bridge.” In one condition, the avatars stood on two different bridges parallel to each other. The bridges were oriented along participants’ facing direction (**Figure 1B**). In the other condition, avatars stood on the same bridges that were orthogonal to the facing direction of the participant (**Figure 1C**). During Experiment 2, the participants were placed on a virtual platform themselves to prevent fear of heights. However, this platform was only visible when participants looked down deliberately.

#### Design and Analysis

We used a 2 (facing direction) × 6 (gap between avatars) × 4 (distance to the subject) × 2 (placement of male and female avatar) × 2 (number of bridges) fully balanced within design. In this experiment, 192 trials were presented twice, resulting in 384 trials. Inclusion of avatar sex (i.e., their position) or bar length in the analyses did not change the pattern of the reported results.

### Results

In Experiment 2 participants judged again distances between facing and non-facing avatars. In this experiment avatars were presented on the same bridge or on two parallel bridges. If the object orientation effect is due to predictive coding we expected no effect of facing direction in the condition with two bridges.

In Experiment 2, participants showed the same pattern of error for the factor facing direction as in Experiment 1. Participants judged the gap between avatars as smaller when the two avatars faced each other and as bigger when they looked in opposite directions, *F*(1,19) = 45.01, *p* < 0.001. **Figure 2** shows the estimated distances depending on avatar facing direction and width of gap between avatars. This corresponds to the results from Experiment 1.

For error, we also observed a main effect of gap width, *F*(1.19,22.57) = 13.24, *p* = 0.001, just as in Experiment 1.

We found a main effect of distance between participant and avatars, *F*(1.37,25.95) = 27.58, *p* < 0.001. The larger the distance was, the larger participants estimated the gap width. Furthermore, we observed an interaction between gap and facing direction, *F*(5,95) = 2.39, *p* = 0.04, and an interaction between gap and distance, *F*(5.98,113.54) = 2.33, *p* = 0.04. A three-way-interaction between gap, bridge and distance originated, *F*(15,285) = 1.92, *p* = 0.02. These effects showed variations of the described main effects under levels of the other factors, which, however, did not reverse any of the described main effects. Neither of the last two interaction was observed in Experiment 1, thus, their reliability can be challenged. Finally, the direction of the pattern did not reveal any unambiguous pattern and is thus not discussed any further.

Contrary to predictions from predictive coding, the interaction of facing direction and number of bridges was not significant, *F*(1,19) = 0.01, *p* = 0.94. No other main effects or interactions attained significance.

### Discussion

Experiment 2 replicated the main effect of facing direction of Experiment 1: participants judged avatars as closer when they faced each other than when they faced away. As this effect was shown on exocentric, not egocentric distance, explanations based on preparation for action of the observer (Gibson, 1979; Proffitt, 2006; Witt, 2017) cannot persist.

Another possible explanation rested upon the extrapolation of avatar location in forward direction as a special case of predictive coding (Kilner et al., 2007; Clark, 2013). To prevent forward movement, we placed the avatars on two parallel bridges in Experiment 2. The gap between the bridges blocked any movement toward or away from each other, at least for larger gaps. When the avatars stood on the same bridge, they could move toward each other. When participants considered the whole scene with walkable and non-walkable paths to predicted forward movement, the object orientation effect should have been smaller or vanish completely in the two bridges condition because no movement toward each other was possible. However, the observed main effect of facing direction was clearly not modified by the number of bridges. Consequently, we could not find evidence for predictive coding which considers the whole scene arrangement. A predictive coding account would still explain our effects if it was limited to the avatars themselves and did not include processing of the whole scene layout.

Another potential explanation of the effect might be based on distance distributions of objects facing each other vs. away. Howe and Purves (2005) used this approach for the Müller–Lyer illusion where a line is judged as shorter when the arrows at the end are pointing inwards, than when pointing outwards. Howe and Purves showed that in a picture database inward pointing arrows more often co-occurred with shorter lines and outward facing arrows with longer lines and that the Müller–Lyer illusion might correctly reflect this asymmetrical length distribution within perception. The same logic might also apply to the object orientation effect in distance perception. Humans facing an observer or each other might typically be closer to each other than when facing away, for example, because interaction usually occurs at shorter distances. The observed bias then would reflect the asymmetrical distribution within the world experienced by humans.

Please note that another popular explanation of the Müller–Lyer illusion by centroids (Whitaker et al., 1996) cannot hold for the present experiments. Centroids of the arrow configurations rather than the actual line lengths are used for distance judgments. As the distance between the inwards facing arrows centroids is shorter, thus are the distance judgments. However, the present and previous experiments equalized the distance on perceptual or actual centroid and their distance cannot explain the observed bias.

Maybe the bridges did not show an effect because the option of moving was still given. For the avatars on two different bridges it was possible to move slightly to the front and get closer to each other. Participants could have taken this into account. Furthermore, the scene is quiet complex and people may not have evaluated all restrictions of the environment. Consequently, they did not reject the possibility of reaching each other although the avatars were on separate bridges. An improvement of the experiment would be to block movements completely and more obviously, for example, by tying the avatars up.

Just like in Experiment 1 we observed a regression to the mean. Small gap widths were overestimated, large gap widths underestimated. This again suggests that participants combined information from the current stimulus and the mean of the presented stimulus range within their judgments (Petzchner et al., 2015).

We also observed a main effect of distance which meant that participants estimated the distance between avatars as larger the further the avatars themselves were located away from participants. This was not observed in Experiment 1. Contrary to Experiment 1 we presented a visual scene which included a horizon. But the horizon in Experiment 2 appeared lower in the field of view than when standing on a ground plane because the observer and the avatars were lifted up on bridges. According to the phenomenon of relative height people judge objects with the same retinal size bigger the closer they are lifted toward the visual horizon (Ozkan and Braunstein, 2010). Due to the visual elevation avatars could have been perceived as further away, the more so the further away they were from the participant as for shorter distances additional visual depth cues such as stereovision lead to better distance estimates that is less distance overestimation. As the Ponzo illusion shows (Ponzo, 1910) the same retinal size (inter-avatar distance) perceived at further distance from an observer will also be perceived as larger. Consequently, an increased overestimation of the observer-avatar distance could have resulted in larger perceived inter-avatar distances.

In summary, with two experiments we showed that the object orientation effect for the perception of distances (Takahashi et al., 2013; Jung et al., 2016; Foster et al., unpublished) is not limited to egocentric distances, but also occurs in case of exocentric distances between avatars. Therefore, the effect does not rely on action preparation. Participants might have applied a strategy of using the shortest horizontal line fitting between the avatars rather than taking the distance between the perceptual avatar midline. This strategy of the shortest line, however, could not explain results from previous experiments with egocentric distances. We tested another possible explanation of the object orientation effect, namely predictive coding. The variation of the plausibility of avatar movement by having the avatars connected by a walkway vs. not connected did not alter the observed effect. Therefore, an interpretation of predictive coding which takes the whole scene layout into account seems unlikely in our case. Nevertheless, we cannot reject the theory of predictive coding as an explanation of the object orientation effect. The judgment bias might also reflect natural statistics on objects facing each other vs. away from each other. Further experiments are required to understand the underlying cause of the object orientation effect better.

## Ethics Statement

This study was carried out in accordance with the recommendations of ethics committee of the University Clinic Tübingen with written informed consent from all subjects. All subjects gave written informed consent in accordance with the Declaration of Helsinki. The protocol was approved by the ethics committee of the University Clinic Tübingen.

## Author Contributions

MW implemented the experiments and collected and analyzed the data. MW wrote the manuscript with support of TM. All authors conceived of the presented experiments, discussed the results, and contributed to the manuscript.

## Conflict of Interest Statement

The authors declare that the research was conducted in the absence of any commercial or financial relationships that could be construed as a potential conflict of interest.
